# Effects of a Teaching Personal and Social Responsibility Model Intervention in Competitive Youth Sport

**DOI:** 10.3389/fpsyg.2021.624018

**Published:** 2021-03-05

**Authors:** Federico Carreres-Ponsoda, Amparo Escartí, Jose Manuel Jimenez-Olmedo, Juan M. Cortell-Tormo

**Affiliations:** ^1^Department of Sport Sciences, Faculty of Education, University of Alicante, Alicante, Spain; ^2^Department of Basic Psychology, Faculty of Psychology, University of Valencia, Valencia, Spain

**Keywords:** positive youth development, sport competition, teaching personal and social responsibility model, prosocial behaviors, self-efficacy

## Abstract

The aim of this study was to implement the teaching personal and social responsibility (TPSR) model in a competitive context analyzing the differences between the intervention and the control group on personal and social responsibility, prosocial behaviors, and self-efficacy in youth soccer players. Participants were 34 youth soccer players between the ages of 14 and 16 years old (15.18 ± 0.72) divided into two different soccer teams of 17 members, corresponding to the control and intervention groups. The implementation of the TPSR model took place during 9 months, including initial and ongoing coach training (3 months), program implementation (three sessions per week lasting 90 min during 6 months), and a series of expert-led seminars for athletes (one session per week lasting 90 min during 4 months). The questionnaires used to collect data were the Personal and Social Responsibility Questionnaire, Prosocial Behavior Scale, and two Children’s Self-efficacy Scales. Results indicated that the TPSR intervention group obtained an increase in post-test levels of personal and social responsibility, prosocial behavior, and self-efficacy due to the application of the TPSR model compared with control group that used a conventional sport teaching methodology. The conclusion is that the TPSR model has the potential to be adapted and implemented with flexibility in youth sport competition contexts in order to improve personal and social responsibility, prosocial behavior, and self-efficacy.

## Introduction

Traditionally, some researches have been considering sport as the perfect instrument to promote positive development in childhood and adolescence (Petitpas et al., 2005; Bailey, 2006; Holt et al., 2017). Positive youth development (PYD) has been considered as a valuable approach to understand youth’s developmental process in a vast array of settings such as sport (Damon, 2004; Lerner et al., 2009; Shek et al., 2019). PYD focuses on a growth process through which adolescents go from being cared for, to taking greater responsibility for the control of their lives, their own well-being, and the well-being of others (Lerner et al., 2009). Hence, it remains critically important to understand that competitive youth sport can no longer be assumed that contributes to PYD outcomes simply through its participation (Strachan et al., 2011). Previous research has revealed how participation does not lead to a uniform experience for all involved, based predominantly on the level of competition experienced and the type of coaching received (Petitpas et al., 2005; Camiré, 2014; Côté et al., 2014). There are recent approaches arguing that youth sport talent development and positive youth development are compatible and mutually beneficial, given that both involve a focus on empowering personal and social strengths (Harwood and Johnston, 2016; Jørgensen et al., 2019). In order to achieve these positive outcomes, competitive youth sport programs must be well-designed with educational objectives, implemented and evaluated to ensure the best aspects of sport experience (Bailey, 2006; Gould and Carson, 2008; Turnnidge et al., 2014; Holt et al., 2017; Santos and Martinek, 2018; Camiré and Santos, 2019). Moving forward, to genuinely promote the educational potential of competitive youth sport, Santos and Martinek (2018) establish four specific strategies. First, that each coach assumes the double objective of improving sport skills of their athletes and at the same time, helping them to learn life lessons and build positive character through sports sessions. Second, converting PYD into specific behaviors, adapted to the athletes’ ages, so that they can recognize, evaluate, and transfer these life skills to other areas of their lives. Third, progressively integrate a PYD-based approach into training sessions, starting from the coach’s reflection on his/her teaching model and pedagogical approaches in order to recognize whether it favors reflection, decision-making, leadership, and explicit transfer of learning behaviors. Fourth, maintain a balance between the expectations of winning on the one hand, and on the other, systematically intervene in the PYD regardless of the demands of the sporting season. In this sense, Santos et al. (2019) recently called for more recommendations that explore coach education programs in order to help youth sport coaches overcome challenges with PYD principles and strategies in their coaching practices. It is also necessary to better understand how competitive youth sport coaches can foster PYD and performance success to study what factors are relevant for coaches to improve their ability to promote PYD (Allan et al., 2018; Preston et al., 2019).

There is an extensive body of literature that focuses on sports programs for PYD (Gould and Carson, 2008; Camiré et al., 2011, 2018; Kendellen and Camiré, 2019; Strachan et al., 2020) and one of the pedagogical models that has been used successfully for 40 years to foster PYD through physical education (PE) and sport is the teaching personal and social responsibility model (TPSR model; Hellison, 1985, 2011). The TPSR model (Hellison, 1985, 2011) was originally developed to be implemented in sports or physical activities to promote values and teach responsibility to young people and adolescents at risk of social exclusion. The goal of the program was to provide these young people with learning opportunities that would allow them to develop competencies and skills that would help them solve the different challenges in their lives. In this way, the TPSR-based programs focused on providing these opportunities integrating responsibility into physical activity, empowering young people gain self-directed learning skills, building strong instructor – participant relationships and promoting transfer of responsibility (Hellison, 2011). The goals and means of TPSR are in line with social psychology theories, particularly self-efficacy theory (Bandura, 1997), with theories from sport and with aspects of positive youth development (Damon, 2004; Lerner et al., 2009; Shek et al., 2019).

The TPSR model is based on three key elements. The first are the levels of responsibility that athletes must learn to become competent adults: (1) respect of the rights and feelings of others, (2) effort and cooperation, (3) self-direction, (4) helping others and leadership, and (5) transfer of responsibility outside the gym (Hellison, 2011). These levels of responsibility are learned in a learning atmosphere in which the instructor integrates the learning of the levels of responsibility with the sports or PE content. The second key element of the TPSR model are nine specific teaching strategies that teachers or coaches must implement during the program: (1) Modeling Respect, be an example of responsibility by acting and communicating in a respectful way both with each athlete individually and with the whole group; (2) Setting Expectations, make explicit to athletes what you expect of them, in relation to sport-specific goals and skills, as well as attitudes and behaviors; (3) Opportunities for Success, structure the sport session so that all athletes have the opportunity to participate in activities successfully and feel that they are progressing regardless of their skill differences; (4) Fostering Social Interaction, introduce games and challenges in the sports session that favor cooperation, teamwork, and problem solving; (5) Assigning Responsibility, distribute responsibilities or specific tasks to athletes that facilitate the organization of the sports session or some aspect of task management; (6) Leadership, allow athletes to assume leadership roles in some part of the sports session, showing their skills, being in charge of a group of teammates…; (7) Giving Choices and Voices, involve athletes in individual choices and group dialogues, asking their preferences, sharing opinions…; (8) Role in Assessment, allowing athletes to play a leading role in the evaluation of the sports session as well as the evolution of the teaching-learning process; and (9) Transfer, offer athletes examples and challenges on how to transfer attitudes, skills, and healthy lifestyle habits from sport to other areas of their lives. The third key component of the TPSR model is the lesson format. When implementing the TPSR model, it is necessary that each session maintain the same structure, regardless of the levels of responsibility and the physical-sports content. In this way, athletes have clear expectations to better respond to challenges and progress more quickly throughout the entire process. The basic lesson format comprises: the awareness talk, physical activity plan, and reflection time. (a) Awareness Talk, at the beginning of each session (no more than 5 min), it is important to share information with the athletes about the objectives and activities planned for that session and (b) Physical Activity Plan, this includes the majority of the sporting tasks of the session which are all intentionally integrated with the attitudes and behaviors of responsibility through the TPSR instructional strategies. The last minutes are dedicated to reflection time, before the end of the session, all the participants, in a circle around the instructor, reflect on whether the responsibility objectives of the day have been met and the participants can express their opinion and evaluate the group and themselves.

It is unquestionable that the TPSR model has been implemented in different contexts and in several countries (e.g., United States, Canada, New Zealand, South Korea, and Spain), obtaining very promising results. An initial review of 26 studies found that in 19 studies, the TPSR model improved respect, effort, autonomy, and capacity for leadership among athletes and school PE students (Hellison and Walsh, 2002). Another review carried out by analyzing the TPSR model-based programs applied in United States and Spain found that the TPSR model has contributed to the positive development of the children and young people by improving responsibility behaviors, social skills, class environment, and self-efficacy (Caballero-Blanco et al., 2013). Another systematic review of 22 studies on TPSR-based programs in PE setting concluded that successful implementation of TPSR contribute to a range of positive behavioral, social, emotional, psychological, and educational outcomes (Pozo et al., 2018). Related to the TPSR implementation in after school setting, a recent systematic review in which 27 papers were selected, 13 of them provided significant experiences and had a positive impact on staff and youngsters who engaged in sports activities (Baptista et al., 2020). Finally, systematic review of 35 studies classified according to the methodology used shows that the application of the TPSR has found positive results in all the studies analyzed (Sánchez-Alcaraz et al., 2020).

Teaching personal and social responsibility interventions have shown a positive improvement in self-efficacy, prosocial behaviors, and personal and social responsibility (Escartí et al., 2005, 2010, 2012, 2013; Alcalá et al., 2019; Valero-Valenzuela et al., 2019). Self-efficacy refers to “belief in one’s capabilities to organize and execute the courses of action required to produce given attainments” (Bandura, 1997, p. 3). A major challenge faced by youth athletes is the acquiring of a sense of personal agency and self-efficacy (Zimmerman, 2006). Adolescents with a robust sense of efficacy for coping with its unique stressors and interpersonal demands in sport and other key life domains are more likely to face challenges in a persevering and relatively anxiety-free manner (Bandura, 1997; Caprara et al., 2004). Another body of research has found that after implementing the TPSR model in outdoor activities in young people, significant improvements were obtained in prosocial behaviors (Caballero Blanco, 2015a; Manzano-Sánchez et al., 2019). Prosocial behaviors refer to voluntary actions that intentionally benefit others (Eisenberg et al., 2015). High prosociality has been associated with greater peer acceptance and academic achievement and it plays an essential role in creating positive emotional bonds with others and maintaining well-being (Warneken, 2016). Finally, TPSR model highlights the construct of responsibility as a fundamental resource in the field of positive development. The model understands responsibility as a position or moral obligation with respect to oneself and others and presents five levels of responsibility that adolescents and young people must learn to become adapted and efficient people in their social environment (Escartí et al., 2005; Hellison, 2011). Many interventions in the model have confirmed an improvement in the personal and social responsibility of young people in PE classes (Escartí et al., 2010; Fernández-Río and Méndez-Giménez, 2016). As has been shown in previous research, it can be affirmed that each of the variables described, prosocial behavior, self-efficacy, and personal and social responsibility are important psychological resources to be incorporated in youth sport competition programs and clubs (Lee and Choi, 2015; Walsh et al., 2015; Wright et al., 2016; Jacobs and Wright, 2018).

The majority of the studies carried out in the last two decades have implemented the TPSR model during *PE classes* (Cutforth, 1997; Escartí et al., 2010, 2012, 2013, 2018; Hellison, 2011; Wright and Irwin, 2018; Wright et al., 2018; Camerino et al., 2019), in *after-school sports programs* (Cecchini et al., 2003; Martinek and Hellison, 2009; Lee and Choi, 2015; Walsh et al., 2015; Wright et al., 2016; Jacobs and Wright, 2018) *and sport adventure programs* (Stiehl, 2000; Hansen and Parker, 2009; Caballero Blanco and Delgado-Noguera, 2014; Bean and Forneris, 2015; Caballero Blanco, 2015b). However, almost no research on the TPSR model has been implemented in competitive sports contexts. Numerous authors specialized in TPSR and Hellison (2011) himself point out that youth sports competition is a perfect setting to implement the TPSR model, due to the small number of participants and because it is a voluntary access activity. On the other hand, despite the fact that many studies on the TPSR model have been carried out in the last two decades, few studies use a rigorous methodology and lack of well-designed and reported randomized controlled intervention studies. Therefore, the aim of this study was to implement the TPSR model in a competitive context analyzing the differences between the intervention and the control group on personal and social responsibility, prosocial behaviors, and self-efficacy in youth soccer players. Our hypothesis was that athletes who experience the TPSR model will improve their pro-social behaviors, personal and social responsibility, and self-efficacy compared with the control group.

## Materials and Methods

### Participants

The group selection was non-probabilistic (Patton, 2015). They were selected for accessibility and convenience due to the profitability and ease of availability because both groups had immediate accessibility to intervene and obtain the data in a reliable and rigorous way.

Participants were 34 soccer players between the ages of 14 and 16 years old (15.18 ± 0.72). The intervention group consisted of 17 boys (*SD* = 15.12 ± 0.72) from a soccer club located in a city of Alicante (Spain). The control group was also a 17 boys (*M* = 15.18 ± 0.72) from a football club located in a nearby city. Both clubs compete in the same category of 2nd Regional of Alicante, organized by Valencian Soccer Federation (Spain). In the intervention group, the team’s coach was a 44-year-old man, with a specific level 2 soccer qualification endorsed by the Spanish Soccer Federation. He had 6 years of experience managing soccer youth teams. The control group coach was a 35-year-old man with the same level 2 football qualification and accumulates 5 years of experience coaching different youth soccer teams.

Participants’ eligibility was given by their belonging to two teams of the same sport (soccer), same category (2nd Regional of Alicante), same sex (males), similar age (between 14 and 15 year), and same training structure (both groups train three times a week, an hour and a half per session, plus the weekend game) and for having similar socio-demographic club profiles. An important factor to note is that the competitive level of both teams was very similar. The main objective of the two teams at the beginning of the season was winning the Championship or being among the top three classified. Another of the inclusion criteria was that both group coaches were in possession of the 2nd Coaching Level of the Valencian Soccer Federation (Spain) and they had a minimum of 3 years of experience training young soccer players. The exclusion criteria for soccer players were to attend less than 80% of the sport training sessions during the intervention. Three young people of the intervention group were excluded, two of them because they joined the team with 2 months before the end of the intervention and one because he was seriously injured in the middle of the season and did not continue to attend training sessions.

Recruitment method consisted, in the first place, in selecting several sports clubs with which the main author maintains a close collaborative relationship. They were telephoned to offer them the possibility of participating in the study and it was two clubs that showed the greatest interest and availability. This was followed by an initial meeting with the managers and sport coaches of both clubs in their own sports facilities where the objective and the investigation procedure were presented. Finally, the doubts were answered and an informed consent was signed by the managers and sport coaches of each club to be included in the study. The lead study author spent 3 weeks on this process before starting the intervention.

### Instruments

#### Personal and Social Responsibility

The Spanish validation (Escartí et al., 2011) of the Personal and Social Responsibility Questionnaire (PSRQ; Li et al., 2008) was performed to measure personal and social responsibility of the participants. This questionnaire consists of 14 items distributed in two factors of seven items each: social responsibility (items 1–7 inclusive) and personal responsibility (items 8–13 inclusive and 14). Examples of items are: *I try to push myself even though I do not like the task* and *I set goals for myself*. Participants responded on a 6-point Likert scale, from 1 (totally disagree) to 6 (totally agree). Only item 14 of the PSRQ is formulated in a negative sense. To promote a better understanding of the instrument by the study participants, we made the following adaptations to the context of competitive youth sport: we changed the main statement from “in my Physical Education classes” to “in my sports training sessions” and in the Item 2, we adapt it from “I respect my teacher” to “I respect my coach.” The internal consistency of the total scale, measured by Cronbach’s alpha coefficient, was 0.86 and 0.88 in the pre- and post-tests, respectively. The different subscales were 0.85 and 0.87 for social responsibility and 0.78 and 0.76 for personal responsibility.

#### Prosocial Behavior

The Spanish validation (del Barrio et al., 2001) of the Prosocial Behavior Scale (PB; Caprara and Pastorelli, 1993) was performed to measure prosocial behaviors of the participants. This is a 15 item scale (answer format: often, 3; sometimes, 2; and never, 1), containing five control items. Various items offer a description of a child’s behavior denoting altruism, trust, and agreeableness. The internal consistency of the total scale, measured by Cronbach’s alpha coefficient, was 0.74.

#### Perceived Self-Efficacy

The Spanish validation (Carrasco and del Barrio, 2002) of two Children’s Self-efficacy Scales (MSPSE; Bandura, 1991): Self-Efficacy for Enlisting Social Resources (4 items; i.e., *How well can you get teachers to help me when I get stuck on schoolwork*) and Self-Regulatory Efficacy (9 items; i.e., *How well can you resist peer pressure to do things in school that can get me into trouble*), was performed. Responses were classified according to a 5-point Likert-type scale, anchored by *not well at all* (1) and *very well* (5). Cronbach’ coefficient alpha for the subscales ranged from 0.69 for Self-Efficacy for Enlisting Social Resources to 0.89 for Self-Regulatory Efficacy (Choi et al., 2001).

### Procedure

An initial presentation session was given to the families, athletes, and coach of each group to explain the details of the study. After that, the researchers gave an informed consent for each family to read, indicating the rights and obligations of being part of the study, the intervention procedure, confidential treatment of data, and sessions filming. Finally, the doubts were resolved and each family member gave the signed informed consent to the researchers. This session lasted approximately 1 hour in each group and was held in a meeting room in the sports facilities of each club.

The coach and team for the intervention program were randomly allocated. It was conducted an indirect intervention program, consisting of a training the coach and then the implementation of the program by the coach. The intervention program took place over 9 months divided into three phases: (a) initial and ongoing coach training (3 months; from September–December), (b) coach implementation with their athletes (three sessions per week lasting 90 min during 6 months where the coach implemented the TPSR program; from January–June), and (c) a series of expert-led seminars for athletes (one session per week lasting 90 min during 4 months; from March–June).

The control group did not receive any intervention between the pre-test and post-test. However, there was direct contact between the researchers and the coach, as well as several visits throughout the season to check if the sports sessions were running normally.

Before (and after) the intervention, the participants answered the Prosocial Behavior Scale (Caprara and Pastorelli, 1993), the Multidimensional Scales of Perceived Self-Efficacy (EA1 and EA2; MSPSE; Bandura, 1990, 2001), and the Personal and Social Responsibility Questionnaire (PSRQ1, PSRQ2; Li et al., 2008) in two sessions (during training hours and on the days previously agreed with the coaches on two different days to prevent bias due to the time limitation for questionnaire completion) in a quiet environment during 20 min per session. In the pre-test as in the post-test of both groups, the first author of the manuscript explained how to complete the questionnaires and read the questions in order to ensure of their understanding. The researcher stood all time with them solving possible doubts. Furthermore, the athletes in both groups were informed about the confidentiality of the data, in order to ensure that the answers were as honest as possible.

### Coach Training Course

From the beginning of the research, the importance of the coach role in the instruction part was considered as one of the success factors of the program. That is why, although there is no single correct way to implement the TPSR model, according to Hanna-Mari et al. (2019), requires specific training that allows the systematic application of the key elements of the TPSR. The TPSR training plan was designed in collaboration with the intervention group coach through an initial meeting. This allowed to know his strengths and weaknesses in relation to the predominant sports teaching methodology with which he had been working for a long time. The TPSR coaching training was conducted by a sport pedagogy researcher with 5 years of experience teaching the TPSR model. The training structure consisted of two major phases: (1) *Initial intensive* 25-h course on TPSR principles and methodology (Escartí et al., 2005; Hellison, 2011), divided into five training sessions of 5 h, with an interval of 1 week between each session. All the meetings were organized in a club classroom where the coach and trainers could be in perfect conditions to promote a positive learning climate. The main purpose of this initial training was to show the coach the origin and evolution of TPSR and to delve into the key elements (levels and strategies, structure and application of the session, and strategies for conflict resolution; Escartí et al., 2005; Hellison, 2011).

The objectives and contents of each of the sessions of the coach training course are described below. The first session lesson plan was related to the Foundation of the TPSR Model and included an explanation of the responsibility-based teaching strategies, responsibility levels, and lesson format. Throughout different exercises, the coach was able to connect his own experience and some of his general pedagogical and psychological principles with which he felt identified with the theoretical knowledge of the TPSR. In this way, he found many similarities with his pedagogical model of teaching sport, which although he did not name them as specified in the TPSR, nor did he apply them systematically in his sessions, he did consider them important. The second session lesson plan was related to the four themes that represent the essence of TPSR: athlete – coach relationships, integration, transfer, and empowerment. This session allowed the coach to consider the four thematic objectives as central aspects to the implementation of his sports sessions and to be able to adapt his teaching model toward the delivery of responsibility-based pedagogy. The third and fourth session lesson plans were about practical applications of the key elements of the TPSR. Throughout video analysis of different expert TPSR trainers, the coach could become aware of and analyze how to effectively apply the responsibility-based teaching strategies, responsibility levels, and lesson format. Especially in session 4, the coach was introduced and trained with the Tool for Assessing Responsibility-based Education (TARE; Wright and Craig, 2011) and TARE 2.0 (Escartí et al., 2015). To promote a better understanding of the TARE and TARE 2.0 to the context of competitive youth sport of our study, we made a simple semantic adaptation changing the words changing the names teacher for “coach” and student for “athlete.” The same structure was maintained to assess the relationships between results of coach and athletes observations. Finally, TARE 2.0 (Escartí et al., 2015) was used throughout the intervention program as a post-coaching reflection and as strategy to reinforce effective TPSR implementation from the video recordings.

In the last session, the coach designed a TPSR-based soccer lesson plan and act out his plan and complete his first teaching try-out applying the responsibility-based teaching strategies, responsibility levels, and lesson format. After each teaching try-out, the researchers give feedback to the coach. Through different inquiry questions, the coach was able to recognize which aspects of the TPSR he was most familiar with and with which he needed more reinforcement. At the end of the initial intensive course, the researchers gave a TPSR Coach Manual, developed specifically for this intervention, based on the work of Hellison (2011) and Escartí et al. (2018), and a set of support videos with specific content examples of the key elements of the TPSR with the aim that the coach could consult it at any time as well as a way to reinforce the effective implementation of the program. The TPSR Coach Manual consisted of two parts, a first more general, recalling the foundations of the TPSR model and a second more specific part with practical applications. The specific strategies that the coach could use to develop each of the levels of responsibility were shown, as well as a series of instruments and evaluation procedures to confirm the attitudinal progress of the athletes (in relation to TARE 2.0). In addition, a series of concrete actions were established in the lesson format section as a checklist to provide the coach with all the general and specific methodological strategies of the TPSR to integrate into his training sessions. Finally, communication strategies were provided to establish a positive emotional relationship with athletes and resolve conflicts peacefully, as well as a section with examples of sports activities and challenges to explicitly transfer the learning of responsibility to other areas of the life of athletes.

(2) *Continuous training*, after the initial training was completed, the coach began to implement the intervention program in the training sessions. Throughout the implementation process, contact with the coach was continuous. Every 15 days, the main researcher met with the coach, in order to analyze and deepen on the most notable aspects in their work dynamics and the type of contingencies he had encountered. First, the principal investigator recorded the training session and, upon completion, viewed it with the coach, using TARE 2.0 to discuss implementation, identify obstacles, and answer questions about TPSR principles, methodology, key elements, and strategies for conflict resolution. In addition, there was full daily availability of the principal researcher *via* telephone or email. The main objective was to continue with the coach mentoring and supervision to ensure the correct implementation of the TPSR and respond to the coach’s questions and needs (Escartí et al., 2012, 2018; Manzano-Sánchez et al., 2019).

### Intervention Program

The objective of the TPSR model was to guide athletes learn and practice behaviors and attitudes that will help them taking responsibility for their personal well-being and contributing to the well-being of others (Hellison, 2011). The intervention program was structured in collaboration with the coach through four phases: (1) *daily TPSR session format*, (2) *Level 1 (Respect) and Level 2 (Participation and effort)*, (3) *Level III (Self-direction) and Level IV (Leadership and caring)*, and (4) *Transfer*. The order of each phase is due to the criteria based on the coach personal strengths, giving priority to the elements of the TPSR in which he had greater command and confidence after the training program. In this way, the coach kept his motivation high in implementing the program from the beginning. Progressively, the rest of the elements were included to improve their competence and effectiveness. Each phase allowed the systematic application of TPSR within the soccer sessions, as well as enable athletes to integrate responsible behaviors as they move through the sport season.

### *Phase One*: The Daily TPSR Session Format Implementation

In phase one, the coach focused on adapting the training sessions to the daily TPSR format. The soccer lessons were divided into five parts: relational time, awareness talk, physical activity, a group meeting, and reflection time. In *Relational time*, the coach used the minutes before starting the session to establish friendly and informal conversations with the athletes with the aim of building positive relationships and getting to know each athlete individually. Some examples of this consisted of asking them about family aspects, their academic performance, as well as simply asking how they felt that day. This was followed by the *Awareness talk* (5 min), in which the coach gathered the athletes in a circle and shared with them the attitudes and behaviors related to the level of responsibility and the physical-sports objectives of the session. It also served to remember attitudes and behaviors, both negative and positive, that occurred in previous sessions and that are considered relevant to reflect on. This part used to take place in the locker room, as it was a more controlled environment free of interference, in which only the coach and the athletes were present. The responsbility and physical-sports objectives of the session were posted on the walls of the locker room in order to facilitate the understanding and assimilation of them by athletes and resolve any doubts in this regard. The third part was *Responsibility in action* (75 min). In this part, the coach operationalized the responsibility levels through the methodological strategies of the TPSR to help athletes to experience responsibility behaviors while practicing the technical-tactical soccer skills set for that day. The fourth part, *Group meeting* (5 min), the coach allowed time for all athletes express their opinions, make suggestions, ask questions, evaluate the group’s behavior, and to share their perceptions and feelings about the lesson development and show examples of how to improve in the following sessions. Finally, in the last part, the *Reflection time* (5 min), the coach gave the athletes the opportunity to evaluate their own attitude, behavior, or development, and reflect and discuss how to transfer the practiced skills to other settings.

The greatest difficulties the coach had when applying the lesson format were in *Awareness talk*, having a disorderly speech that sometimes exceeded 10 min. With the ongoing training, the coach learned to be clear and concise in establishing the objectives and expectations of the session. Another aspect of difficulty in the first 2 months of the intervention was in the *Responsibility in action* part, especially because he did not offer strategies to provide progressive autonomy and leadership to the athletes in the different activities of the session. Finally, another aspect of difficulty was in the *Group meeting* since the coach used to monopolize the conversation. As the intervention progressed, the coach learned to give more and more voice to the athletes and listen to their opinions.

### *Phase Two*: Level 1 (Respect) and Level 2 (Participation and Effort) Implementation

At the same time that the coach began applying the daily session format, the coach was encouraged to operationalize Level I and Level II. By starting with level 1 (respecting the rights and feelings of others), it was possible for the coach to achieve a greater positive climate in the team and solve the conflicts that were appearing in a peaceful way. The main strategies applied at this level were related to communication skills, such as listening to the coach and teammates when they are speaking, asking for a turn to speak and using non-violent communication despite the conflicts that could arise. Another series of specific strategies to promote respect were: minimizing selfish actions, especially in goal celebrations, and reinforcing positive respectful behaviors shown by athletes during the session. Related to level 2 (participation and effort), the coach continued implementing activities and strategies in order for athletes to actively participate in the activities proposed even when they are not easy or to their liking. Changing groups and providing at least one constructive feedback to all athletes during the sessions were strategies that contributed to increase their intrinsic motivation. The coach was aware that not all athletes should be held to the same standard. Instead, he used to minimize criticism and use instruction and encouragement to help athletes recognize their mistakes and show effort and improvement. It took him 3 months to focus on the level 1 and level 2 intervention.

### *Phase Three*: Level III (Self-Direction) and Level IV (Leadership and Caring)

The implementation of Level 3 (self-direction) allowed the athletes to set short and long term goals, reflect on and evaluate their own progress honestly. The coach started offering different periods of time during the sport session for the athletes to work independently and productively on their own improvement goals. Some specific strategies that the coach began to use more regularly were asking and recognizing athletes’ feelings and perspectives and structuring reward systems thoughtfully that allowed more opportunities for athletes to demonstrate initiative and autonomy behaviors. This allowed them to make responsible decisions about using the practice time correctly, to organize themselves, to choose the sports equipment and the most convenient space, to modify rules, as well as to consult the coach about the personal difficulties they encountered in some tasks and in the practice of soccer skills. Finally, the coach started to implement level 4 (leadership and caring) from the fifth month. This level meant a radical change in team dynamics by showing helpful attitudes and behaviors and leadership roles. The coach was amazed at how the athletes takes seriously the role of teaching an activity or skill to the team, something he had never experienced before, due to fear of losing control of the group.

### *Phase Four*: Level V (Transfer) Implementation

The coach began to implement Level 5 (transfer) from the first moment with level 1 and in parallel with the rest, with the intention of transferring everything learned in each sport session to other significant contexts in athletes lives, such as their family, friends, school, or their neighborhood. At the beginning of the intervention, the transfer was carried out in a more implicit way using the nine methodological strategies to promote the learning of personal and social responsibility: (1) modeling respect, (2) setting expectations, (3) providing opportunities for success, (4) fostering social interaction, (5) assigning management tasks, (6) promoting leadership, (7) giving choices and voices, (8) involving participants in assessment, and (9) addressing transfer of life skills, that allow creating temporary consistency in the implementation of the program, incorporating the four methodological pillars in the sessions and working in an integrated way the different levels of responsibility. After the first 5 months of implementation, the coach was also able to work on the transfer in a more explicit way by offering challenges and examples of how athletes could apply the TPSR levels behaviors in their daily lives. Another explicit strategy was through interviews with the athletes in which he encouraged them to resolve conflicts in the family, groups of friends, and high school applying the behaviors of responsibility learned in the program and checking the effect they had. In the Relational time, especially in a private way, and in the Awareness talk, if it was agreed with the athlete, these transfer challenges were shared and applauded by the rest of the team. The TPSR intervention program was implemented in each of the three weekly sports training sessions, lasting 90 min per session during 6 months.

In the control group intervention, the coach used a conventional teaching methodology in which there was no intentional training or additional contribution from the researchers of the study. This methodology had a classic session structure and was differentiated into three parts (warm-up, main part, and return to calm). However, the sport contents were the same for both the control and experimental groups related to soccer skills and tactics.

### Workshops for Athletes

The research on transference of TPSR model goals is scarce (Santos et al., 2020) and the transfer analysis from the competitive sport context is non-existent. However, Jacobs and Wright (2018) as well as Bean et al. (2018) propose models to facilitate the understanding of the transfer of life skills and strengthen the strategies to offer examples, challenges, and opportunities to promote positive youth development explicitly through a sport program. Following the indications of these authors, the researchers of the present study considered that the 2–3 min that the coach could dedicate to explicitly transfer the responsible behaviors of the training sessions to other domains of the athletes’ lives would not be enough. That is why, a program of workshops was created in addition to the implementation of the TPSR model in the training sessions by the coach with the purpose of promoting the learning of attitudes and behaviors related to levels of responsibility, as well as the transfer of these learnings. These workshops provided athletes with tools that enable them to practice reflective awareness, metacognition, mindfulness, and insight as the fundamental skills that can facilitate the life skill transfer process. According to the educational psychology literature, Jacobs and Wright (2018) suggest that rather than directly teaching life skills *per se*, it may be useful to teach the cognitive strategies that enable athletes to build their own life skills. In this sense, the contents of the workshops offered a set of practical cognitive strategies that enable athletes to improve the understanding and application of personal and social responsibility skills in sports, in academics, in family and social relationships, in order to optimize adolescents’ ability to make decisions about present and future well-being in each of the important areas of your life. The methodology was participatory and experiential, through different methods and techniques such as video forum, role playing, which allowed adolescents to experience and internalize the values and attitudes of the TPSR and challenge themselves to apply them in other areas of their lifetime. A total of 12 seminars were completed, over 4 months, which took place 1 day a week, each session lasting 90 min. Attendance at the workshops was voluntary for athletes. The average attendance at the seminars was 15 participants, with a level of commitment of 92%.

### Statistical Analysis

A descriptive analysis was carried out of the results obtained in the scores of each of the participants in the different dimensions of each questionnaire, according to the moment of obtaining the data (pre-test and post-test). The 95% confidence interval was also calculated. The Shapiro-Wilk normality test established a normal distribution for all the study variables analyzed. To find out if there were differences between the study groups at the beginning of the intervention, a MANOVA test was used. For the multifactorial ANOVA, the group variable (intervention/control) and the test variable (pre-test and post-test) were used as independent variables. The scores obtained from each of the analysis dimensions of the test questionnaires were taken as dependent variables. The calculation of the effect size was carried out by calculating Eta-square (*η*^2^), establishing the scale small (0.02), medium (0.13), and large (0.26; Cohen, 1988).

## Results

Table 1 shows the descriptive data of the results obtained for pre-test and post-test in each of the dimensions analyzed in the study. In MANOVA test, multivariate contrasts indicate that there are differences in the interaction of the dependent variables, according to the group to which they belong (intervention or control; Pillai’s Trace, *F* = 10.341; *p* < 0.001; *η*^2^ = 0.46), thus as the moment in which the test was performed (pre-test/post-test; Pillai’s Trace, *F* = 7.17; *p* < 0.001; *η*^2^ = 0.37). In the same way, differences are also established between the interactions of the independent variables analyzed (Group * Test; Pillai’s Trace, *F* = 7.81; *p* < 0.001; *η*^2^ = 0.39).

**Table 1:** Descriptive data.

	Pre-test						Post-test					
	Intervention			Control			Intervention			Control		
Dimensions	Mean	SD	IC (95%)	Mean	SD	IC (95%)	Mean	SD	IC (95%)	Mean	SD	IC (95%)
PB	13.88	2.44	12.62–15.14	13.94	2.48	12.66–15.22	16.12	0.69	15.76–16.48	14.00	1.83	13.06–14.94
PSRQ1	35.18	4.53	32.85–37.51	34.71	4.18	32.56–36.85	37.76	1.52	36.98–38.55	34.65	3.35	32.92–36.37
PSRQ2	34.76	2.84	33.30–36.32	34.47	2.21	33.33–35.61	37.82	1.07	37.27–38.38	34.06	1.71	33.18–34.94
EA1	16.53	1.66	15.67–17.38	16.35	1.16	15.75–16.95	19.24	0.83	18.81–19.66	16.41	1.37	15.71–17.12
EA2	22.12	2.39	20.89–23.35	21.88	2.78	20.45–23.31	23.88	0.85	23.44–24.22	22.12	1.83	21.18–23.06

Mean, arithmetic mean of the scores; SD, standard deviation; 95% CI, 95% confidence interval; PB, prosocial behavior; PSRQ1, personal responsibility; PSRQ2, social responsibility; EA1, self-efficacy for enlisting social resources; EA2, self-regulatory efficacy.

The between-subject analysis indicates that there are differences in the dependent variables, since for the group dependent variable (intervention or control), differences are established in all the independent variables analyzed. The same occurs when the effect of the independent variable test (pre-test/post-test) is studied, where significant differences were also recorded for all the dependent variables of the study.

In the interaction of the two independent variables (group * test), it is established that for the dependent variables prosocial behavior (PB; *p* = 0.028), social responsibility (PSRQ2; *p* = 0.001) and Enlisting Social Resources (EA1; *p* < 0.001), significant differences were reported. Thus, the implementation of the TPSR carried out in youth soccer players shows significant differences for those athletes who received the intervention of the program, vs. those who did not receive it for each of these dependent variables (Table 2).

**Table 2:** Results between-subjects effects.

ID	DV	*F*	*p*	*η*^2^
Group	PB	4.49	0.038	0.066
	PSRQ1	4.24	0.043	0.062
	PSRQ2	16.43	<0.001	0.204
	EA1	22.81	<0.001	0.263
	EA2	3.87	0.050	0.057
Test	PB	5.58	0.021	0.080
	PSRQ1	2.11	0.151	0.032
	PSRQ2	6.98	0.010	0.098
	EA1	19.37	<0.001	0.232
	EA2	3.871	0.050	0.057
Group*Test	PB	5.024	0.028	0.073
	PSRQ1	2.31	0.133	0.035
	PSRQ2	12.01	0.001	0.158
	EA1	17.76	<0.001	0.217
	EA2	2.26	0.014	0.034

Mean, arithmetic mean of the scores; SD, standard deviation; 95% CI, 95% confidence interval; PB, prosocial behavior; PSRQ1, personal responsibility; PSRQ2, social responsibility; EA1, self-efficacy for enlisting social resources; EA2, self-regulatory efficacy; ID: independent variable; DV, dependent variable; *F*, *F* value; *p*, *p* value; *η*^2^, eta square.

Finally, the independent variables Self-efficacy for Enlisting Social Resources (EA1) and Social Responsibility (PSRQ2) showed a medium effect size (*η*^2^ = 0.21 and *η*^2^ = 0.15, respectively). For the variables Prosocial Behavior (PB; *η*^2^ = 0.07), Personal Responsibility (PSRQ1; *η*^2^ = 0.03), and Self-Regulatory Efficacy EA2 (*η*^2^ = 0.03), a small effect size was recorded.

## Discussion

The purpose of this study was to analyze the effects of a TPSR model-based program implemented in a sport club context to assess its effects on responsibility, prosocial behaviors and self-efficacy. This study addresses the need to investigate the effects of the implementation of the TPSR model in competitive sports contexts and offers guidelines to continue providing evidence in this sporting context. Overall, intervention group obtained an increase in post-test levels of personal and social responsibility, prosocial behavior, and self-efficacy due to the application of the TPSR model compared with control group that used a conventional sport teaching methodology. These positive effects found after the implementation of the program are consistent with those of other research on TPSR interventions in sport settings (Hellison and Walsh, 2002; Escartí et al., 2012, 2013, 2015, 2018; Gordon et al., 2016; Wright et al., 2016).

In previous studies, prosocial behavior has been found to be a predictor of personal and social responsibility (Gutiérrez et al., 2011). The data of the present investigation agree with other TPSR implementations, that although they have been carried out in other sports contexts as in outdoor activities or activities in school context, prosocial behavior improved significantly after applying the TPSR model also showed that encouraging prosocial behavior positively predict students’ personal and social responsibility (Escartí et al., 2011; Caballero Blanco and Delgado-Noguera, 2014; Manzano-Sánchez et al., 2019). This fact suggests that the strategies related to the TPSR model applied by the coach of the intervention group, such as *offering leadership opportunities*, *giving choices and voices*, and *sharing an active role in assessment* to athletes, have perhaps contributed to an improvement in social skills and communication of athletes, which has had a positive impact on the development of prosocial behavior. Despite the initial difficulties of the coach in the intervention to offer opportunities to athletes to be more active in their learning process collaborating with each other and allowing them to teach an activity or skill to the team, the impact on prosocial behavior has been high. This finding led us to affirm that TPSR model intervention in competitive sports contexts could involve improvements in youth prosocial behavior if the coach use specific strategies related to Level III (Self-direction) and Level IV (Leadership and caring) and other examples of strategies as constructive feedback, supporting, congratulating, and encouraging one’s teammates that contribute to experience a more pleasant sport experience and lead athletes to try harder and perform better (Kavussanu and Stanger, 2017; Pizzi and Stanger, 2019).

As expected in the initial hypothesis, the athletes who participated in the experimental group obtained significant improvements in social responsibility over those in the control group after participating in the program. These results coincide with other investigations that have found an improvement in responsibility behaviors after an intervention program based on Hellison’s responsibility model (Hellison and Walsh, 2002; Gordon, 2007, unpublished; Wright and Burton, 2008; Lee and Martinek, 2009; Hayden, 2010; Caballero Blanco, 2012; Escartí et al., 2012; Bean and Forneris, 2015; Valero-Valenzuela et al., 2019). The situations in which adolescents present higher indices of responsibility are those related to the ability to show respect for others, help others, be kind to others, control impulses, and collaborate with others. On the contrary, the situations in which adolescents feel less responsible are those derived from making an effort even if they do not like the task. This could explain that there is not a significant improvement in personal responsibility factor in the present investigation. This fact suggests that since it is an intervention carried out in a team sport such as soccer, there is a greater predominance of social responsibility over personal responsibility. In this way, the results obtained suggest the importance of developing specific strategies by coaches who use the TPSR model in team sports contexts to specifically contribute to an improvement in personal responsibility. In our case, due to the coach’s lack of previous experience to apply strategies that favor personal responsibility, related to levels II (Participation and effort) and III (Self-direction), it is possible that there has not been a significant increase in this variable. In this sense, Llopis-Goig (2011) indicated that improvements in personal responsibility appeared when the TPSR model was applied for a long time and 6 month duration of our intervention was not enough time to achieve the full learning of responsibility. Another important implication of this results for youth sport competitive coaches is that an improvement in personal responsibility could be achieved through empowerment strategies that promote independence and autonomy, provide opportunities for making choices, share leadership roles, and give athletes voice in the program (Wright et al., 2018).

Related to self-efficacy, we hypothesized that athletes who experience the TPSR model will improve their self-efficacy compared with the control group. The factor that increased the most was Self-efficacy for Enlisting Social Resources (EA1) related to the strategies applied by the TPSR model by the coach, such as modeling with peers, giving power and voice to athletes, offering them feedback on their performance, encouraging autonomy, and strengthening the effort, that have promoted the development of the perception of self-efficacy in athletes of the experimental group. The results of the present investigation agree, despite being in different sports contexts, with those found by Escartí et al. (2010). In this study, the TPSR model was implemented during one academic year in PE classes and the intervention group also experienced an increase in self-efficacy in obtaining social resources in the post-test. Another study that follows a line consistent with the present one and that obtained similar results was carried out by Escartí et al. (2006), in which they applied the responsibility model to a group of at-risk adolescents in PE classes during an academic year. In our study, the coach contributed to the improvement of self-efficacy by applying specific Level 1 strategies (Respect) ensuring a fair environment and other general strategies such as encourage and praise even small successes and provide direct and specific feedback to every player during the training sessions. After implementation, a significant improvement was obtained in the levels of self-efficacy of the experimental group compared to the control group, specifically on self-efficacy in enlisting social resources and self-regulatory efficacy for learning. It has been shown that a low level of self-efficacy can lead not only to a decrease in sports performance, but also to maladaptive behaviors in young people and a lack of motivation and adherence to sports (Reverdito et al., 2017). For this reason, a practical orientation of these results is the importance that youth sport competitive programs strengthen the development of self-efficacy in yungsters and promote skills that allow them to believe in their own abilities (Reverdito et al., 2017; Camiré and Santos, 2019).

Based on the results, youth sports competition programs can no longer be conceptualized as a single coach and have to evolve to include improvement in both athletic performance and PYD that extends beyond the sport domain. The results of the present investigation suggest that the pedagogical model used by the control group coach would not suppose a sufficient stimulus to be able to contribute to the improvement of the study variables and, therefore, to a PYD through the sports program. Therefore, it can be affirmed that the TPSR model allows nurturing the coach-athlete relationship, the development of essential values such as prosocial behavior, self-efficacy, and personal and social responsibility in the context of youth sports competition, which lays the foundation for effective learning life skills.

Some limitations to the study should be noted. One limitation is that only two sports coaches participated in the study and the participants in both groups were only men. This small number of coaches makes it difficult to generalize the results to other coaches who develop their work in other sports contexts and especially, in female practice settings. Future studies should include a broader sample of sports coaches, as well as a greater variety in terms of gender, sport played, and different levels of competition. This research relies on convenience samples, therefore, is another limiting factor for the generalization of the results. An important suggestion is the need to use mixed methodology (including athletes, coaches, and parents perspectives) in future research to reinforce the value of the results and obtain a more comprehensive view of the mechanisms to favor the PYD through competitive youth. Although the coach reported that youth eventually behaved in accordance with the goals of the program during the sport sessions, we do not have results of them as suggested in Wright et al. (2016) and Camiré et al. (2019).

## Conclusion

Findings suggest TPSR model have the potential to be adapted and implemented with flexibility in youth sport competition contexts in order to improve personal and social responsibility, prosocial behavior, and self-efficacy. In our case, although we have been obtained very positive results self-efficacy, prosocial behavior, and personal and social responsibility, it has only been possible to compare them with studies in PE and after-school sport activities (Escartí et al., 2010, 2012, 2018). As Jacobs and Wright (2018) recently suggested, we need more research to understand better the development of life skills youth sport competition programs. Continued study of implementation is important and may be enhanced by the used of mixed methods.

The present study could serve as reference for future investigations applying TPSR model in youth sport competition programs. An interesting avenue for future research should be to examine the relationship between the implementation of the TPSR by the sports coach and the results such as the athletes’ learning of social and emotional learning competencies and other variables such as sports performance, performance academic, and family climate, since these results have previously been associated with effective personal and social development programs (Wright et al., 2018; Camiré et al., 2019). Future applications of TPSR model in youth sport competition should be aimed at intervening in groups of both sexes, from an earlier age and covering the entire adolescent age range. Furthermore, it would be necessary to assess the effect of TPSR in participants with longitudinal studies. A future proposal to consider is to apply the TPSR in all the groups or teams of the same club or sports entity, in order to verify its effects during the sports career of adolescents.

## Data Availability Statement

The raw data supporting the conclusions of this article will be made available by the authors, without undue reservation.

## Ethics Statement

The study was carried out in accordance with the Declaration of Helsinki and was accepted and verified by the Ethics Committee of the University of Alicante, Spain, ID: UA-2020-09-02. Written informed consent to participate in this study was provided by the participants’ legal guardian/next of kin.

## Author Contributions

FC-P and AE developed the project and supervised the design of the study and were responsible for the literature review. FC-P, AE, JC-T, and JJ-O were responsible for the critical revision of the content and the drafting of the manuscript. FC-P, JC-T, and JJ-O collected and codified the data. JC-T and JJ-O collaborated in data analysis and redaction of results. All the authors approved the final, submitted version of the manuscript.

### Conflict of Interest

The authors declare that the research was conducted in the absence of any commercial or financial relationships that could be construed as a potential conflict of interest.
